# smokeSALUD: exploring the effect of demographic change on the smoking prevalence at municipality level in Austria

**DOI:** 10.1186/s12942-016-0066-4

**Published:** 2016-10-07

**Authors:** Melanie Tomintz, Bernhard Kosar, Graham Clarke

**Affiliations:** 1 GeoHealth Laboratory, Department of Geography, University of Canterbury, Private Bag 4800, Christchurch, 8140 New Zealand; 2 Geoinformation and Environmental Technologies, Carinthia University of Applied Sciences, Europastrasse 4, 9524 Villach, Austria; 3 School of Geography, University of Leeds, Leeds, LS2 9JT UK

**Keywords:** Health decision support, Small area modelling, Deterministic reweighting, simSALUD, Austria, Spatial microsimulation, Web-based application, Smoking, Demographic change, Municipalities

## Abstract

**Background:**

Reducing the smoking population is still high on the policy agenda, as smoking leads to many preventable diseases, such as lung cancer, heart disease, diabetes, and more. In Austria, data on smoking prevalence only exists at the federal state level. This provides an interesting overview about the current health situation, but for regional planning authorities these data are often insufficient as they can hide pockets of high and low smoking prevalence in certain municipalities.

**Methods:**

This paper presents a spatial–temporal change of estimated smokers for municipalities from 2001 and 2011. A synthetic dataset of smokers is built by combining individual large-scale survey data and small area census data using a deterministic spatial microsimulation approach. Statistical analysis, including chi-square test and binary logistic regression, are applied to find the best variables for the simulation model and to validate its results.

**Results:**

As no easy-to-use spatial microsimulation software for non-programmers is available yet, a flexible web-based spatial microsimulation application for health decision support (called simSALUD) has been developed and used for these analyses. The results of the simulation show in general a decrease of smoking prevalence within municipalities between 2001 and 2011 and differences within areas are identified. These results are especially valuable to policy decision makers for future planning strategies.

**Conclusions:**

This case study shows the application of *smokeSALUD* to model the spatial–temporal changes in the smoking population in Austria between 2001 and 2011. This is important as no data on smoking exists at this geographical scale (municipality). However, spatial microsimulation models are useful tools to estimate small area health data and to overcome these problems. The simulations and analysis should support health decision makers to identify hot spots of smokers and this should help to show where to spend health resources best in order to reduce health inequalities.

## Background

Smoking is directly responsible for many diseases, sometimes leading to death (worldwide this figure is estimated to be around 10 %). In addition, passive smokers are at high risk of also developing smoking-related diseases [1]. The Austrian Government is well known for offering a generous social support system, including one of the best health care systems in the world. However, the topic of health inequalities has attracted growing attention, both at the European Union (EU) level and in Austria itself. This issue is especially important in the field of health promotion and prevention. An effective resource distribution strategy is required for areas with high demand (e.g. high rates of smoking, obesity, drug addiction) and poor accessibility to health care providers. Health inequalities can be addressed through government actions and policies but need to be identified first. In particular, identifying regional inequalities is essential for the future distribution of government resources. But one of the problems with the official surveys conducted by Statistics Austria is that health related data mainly exists at the federal state level only. This data provides an interesting overview of the health of the nation, but for regional planning purposes these data are often insufficient and provide no reliable estimates below state level. However, spatial microsimulation models are useful tools for estimating small area health data and thus helping to overcome these problems. Many studies have used spatial microsimulation to estimate health care demand [2–4], but in Austria little research exists to date with the exception of the research project SALUD (SpatiAL microsimUlation for Decision support) which focuses on building a spatial microsimulation model for Austria. Within this project a web-based spatial microsimulation application (simSALUD) was developed to estimate, validate and visualize smoking prevalence at the municipality level using deterministic reweighting approaches. Some microsimulation applications exist on the Web [5, 6] but an intensive literature search through current spatial microsimulation frameworks shows that at the moment no easy-to-use web-based spatial microsimulation applications, which includes spatial visualization methods for non-programmers, are available as yet.

This paper focuses on the topic of smoking because smoking is a major risk factor for poor health and premature mortality. As it is based on a poor lifestyle choice, it is in theory preventable. For effective preventive actions at the regional level, it is important to know where high numbers of smokers live and whether significant variations exist in such rates between municipalities. Recent Organisation for Economic Co-operation and Development (OECD) statistics show that 23.2 % of the adult population smoke regularly in Austria, which is 2.2 % above the average across all 41 OECD countries [1]. Austria also tends to follow the general pattern of gender differences across Europe, with higher smoking rates among men (27.3 %) in comparison to women (19.4 %) [7].

The demographic and socio-economic changes between 2001 and 2011 are shown by the census and registered-based census, respectively. According to Statistics Austria, the population count in 2015 was 8,584,926, which is an increase of 209,762 persons (2.4 %) when compared to the population count in 2011. Taking a closer look at population change between 2001 and 2011, there was an increase of 4.2 % (354,218 persons) which was mainly driven by immigration and less by an increase of births over deaths. Out of the nine provinces in Austria, only one province (Carinthia) saw a population decline (2853 persons or 0.5 %). In contrast, population increase was seen across all other Austrian regions. Vienna had the highest increase with 8.7 % (148,899 persons), followed by Vorarlberg with 5.2 % (19,171 persons) and Tyrol with 5.1 % (36,025 persons). An increase in people aged 25–65 with a tertiary qualification was apparent, especially in the age group 27–36 (7.0 %). There was an increase of 2.4 % for the age group 0–19 years; an increase of only 0.1 % for the age group 20–64 years; but an increase of 2.2 % for the age group 65 and older [8].

The aim of this paper is to estimate changes in smoking prevalence at the municipality level between 2001 and 2011 based on population change in Austria. For this purpose, the *smokeSALUD* package is constructed using a deterministic reweighting spatial microsimulation methodology and implemented in a web-based software application called simSALUD,^1^ developed within the funded research project SALUD at the Carinthia University of Applied Sciences in Austria. The simulation algorithm estimates small area data by merging individuals from the national survey data with census data at the municipality level based on common variables (e.g. age, marital status). However, these variables are limited and not comparable between both datasets without additional data processing. This requires careful data manipulation and pre-analyses are required when using survey and census data to build a spatial microsimulation model [9]. Microsimulation is excellent for estimating ‘missing data’, and as data on smoking prevalence is only available at province level in the health survey by Statistics Austria, this is important for estimating small-area smoking rates. The Austrian Health Surveys were conducted in 1973, 1983, 1991, 1999, 2006/07 and 2014, but with a new design making prior studies not entirely comparable. The data for the latest survey, however, was not accessible until this study was finished. With *smokeSALUD* the aim is to explore the spatial variations in smoking at the municipality level at two different points in time, i.e. 2001 and 2011. This information is highly valuable for health policy decision makers to help to understand whether government smoking prevention strategies appear to be working or not. Austria is one of the last countries that will implement a full smoking ban for cigarettes and e-cigarettes in public places from May 2018. So far, there has only been a partial implementation: dividing smoking and non-smoking rooms in bars and restaurants which has caused severe reconstruction costs for many owners in the food and drink industry. Austria also scores lowest on a tobacco control scale in 2007, 2010 and 2013, as was shown by [10]. This latter report also shows that other countries have proven the success of their smoking policy interventions (such as the UK, Ireland or Iceland) and the model introduced in this paper can help identifying needs at a small geographical scale and to model the impact of successful interventions for Austria. In this way we hope the modelling framework has wider international appeal, providing a framework for examining smoking-related policies across the world and helping to target areas less likely to respond to current policies. But before doing so, the geography of the smoking population for small areas needs to be modelled and understood.

## Methods

### Methodology and workflow

Spatial microsimulation models have been built since the 1980s and there are different methods for building such models, including deterministic and probabilistic approaches [11]. There are few freely available software packages to implement spatial microsimulation. For this reason, deterministic approaches have been implemented in simSALUD, a web-based spatial microsimulation application. The advantage of using a deterministic over a probabilistic approach is that the model terminates at a unique solution each time it is run. Therefore, adjustments to the input data are immediately obvious as to whether the changes have improved the model outcome or not. *smokeSALUD* is a static model designed to match large scale survey and small area census data based on common key constraints that are most likely to predict the variable of interest; in this case the smoking population. The process can be seen as a form of cloning exercise, where persons from the survey are matched to each small area, in our study the municipality, based on the selected key constraints. A reweighting algorithm thus calculates how well a person with certain characteristics fits the characteristics of persons known to live in a certain small area [12, 13]. Given key population characteristics we can estimate whether this person is a smoker or not and this information is also reweighted to provide the results for each single municipality. The models for smokeSALUD are run using the Web application simSALUD.

**Fig. 1:**
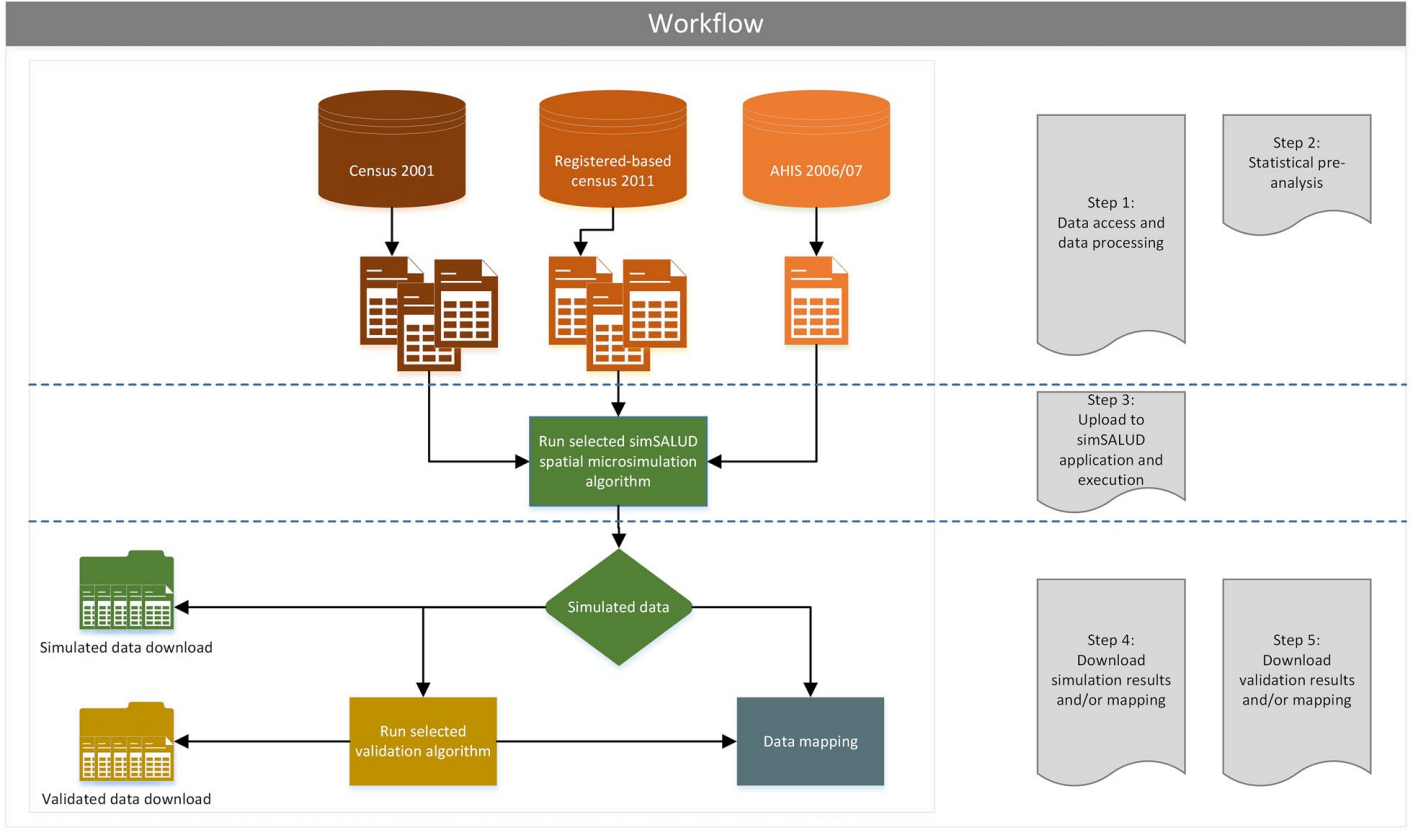
Structure of the spatial microsimulation model using the simSALUD application

The structure of the model process is shown in Fig. 1 and each step is described in detail below. The underlying algorithm is explained elsewhere [14]. The structure can be divided into five steps (data processing, pre-analyses, model execution, validation and the visualization of the simulated output) and is described according to the case study in the next subsections. Steps 3–5 can be executed directly within the simSALUD application, which has been developed by the authors as part of the research project SALUD.

### Step 1: Data and data processing

Three datasets are relevant for this case study. The first dataset is the Austrian Health Interview Survey (AHIS) for 2006/07 which holds variables for demographic, socio-economic and health related information of 15,474 individuals aged 15 or above at the federal state level. The dataset also includes the variable “number of daily smokers”, which is the health variable of interest and (for each person) a weight to compensate for the bias of the survey that corresponds to the total population. The second dataset is the 2001 census for municipalities and the third dataset is the registered-based 2011 census for municipalities. Both census datasets (2001 and 2011) have demographic and socio-economic information concerning the Austrian population but no health related data is included. There are 2379 municipalities in Austria and the 2001 census showed a population of 6,679,444 (people 15 years and over) and the 2011 registered-based census recorded a population of 7,174,250 persons (people 15 years and over). In this first step all datasets are tested for completeness and data cleaning is conducted if required.

### Step 2: Statistical pre-analysis

Statistical analyses prior to building a spatial simulation model are important in order to identify the best predictors (i.e. characteristics of a person) of the variable being estimated—in this case the number of smokers. For this study, the chi-square test, regression analysis and t-test are applied within the statistics software IBM SPSS Statistics 21.0. In addition, an extensive literature review on the influences and characteristics of smokers was undertaken. This is necessary because creating synthetic microdata requires linking datasets based on common variables, but those variables must also be important for estimating the key ‘missing data’. For a detailed discussion of the role of the constraint variables and the choice of constraints in microsimulation see [3, 15, 16]. Previous literature [2, 17, 18] indicates that smoking status is strongly predicted by variables such as age, sex and social status which are available in the AHIS. For the spatial–temporal analysis, it is also necessary to have the same variables in both census datasets (census 2001 and 2011). Otherwise adjustment procedures need to be undertaken between the datasets in order to have the same base population.

The chi-square method is chosen because it can be measured at an ordinal or nominal level (i.e. categorical data). Calculating a statistical correlation method is not possible because this would require measuring both variables at the interval or ratio level (i.e. continuous scale) which is not the case for this study. However, chi-square is used to find a possible relationship between two categorical variables. The measurement parameters for Phi and Cramer’s V, which vary between 0 and 1, show the same value for all variables. The closer the value is to 1, the stronger is the relationship between the dependent and independent variable. The binary logistic regression is used to predict the probability that an observation falls into one of two categories of a dependent variable, based on one or more independent variables that can be either continuous or categorical. The output also calculates the Cox & Snell R-square and Nagelkerke R-square values, which are both methods of calculating the explained variation. Another statistical test applied is the independent samples t-test that compares the means between two unrelated groups on the same continuous, dependent variable. For example, it can be used to understand whether education level differs between smokers and non-smokers.

Tables 1 and 2 show the results for all constraints using the chi-square and binary logistic regression analyses. The values show the results for the final constraints chosen after the pre-analysis process which tested different variable combinations until the best validation results were found. The results in Table 1 show that there is a statistically significant association (p < 0.001) between daily smokers and these chosen constraints, although the strength of association between the variables is weak (Phi/Cramer’s V > 0.5 high association). Phi and Cramer’s V show the highest strength of association between daily smokers and the constraint age (0.240), in contrast to education which shows the lowest value of 0.068 between the final selected constraints.

**Table 1:** Summary of the chi-square analysis relating to the category “daily smokers”

Constraint	Chi square	
	Symmetric measures Phi/Cramer’s V	Asymptotic significance (2-sided)
Age	0.240	p < 0.001
Education	0.068	p < 0.001
Marital status	0.180	p < 0.001
Sex	0.083	p < 0.001
Occupational status	0.186	p < 0.001

**Table 2:** Summary of the binary logistic regression analysis relating to the category “daily smokers”

Constraint	Sub-constraint	Binary logistic				
		B	Significance	Expected (B)	95 % C.I. for EXP(B)	
					Lower	Upper
Age	15 above	−0.115	p < 0.001	0.892	0.876	0.907
Education	University	−1.011	p < 0.001	0.364	0.296	0.446
	With A-level	−0.485	p < 0.001	0.616	0.548	0.691
	Without A-level					
Marital status	Single					
	Married	−0.135	p < 0.017	0.873	0.781	0.975
	Widowed	−0.414	p < 0.001	0.661	0.521	0.837
	Divorced	0.894	p < 0.001	2.446	2.063	2.899
Sex	Male					
	Female	−0.309	p < 0.001	0.734	0.676	0.796
Occupational status	Employees	0.606	p < 0.001	1.833	1.673	2.007
	Employer	0.188	p < 0.030	1.207	1.012	1.438
	Non-employed					

The explained variation through the binary logistic regression test ranges from 7.7 to 11.8 %. The results for each sub-constraint shown in Table 2 can be defined (one example for each constraint) as follows: by an increase of one (5 years in age), the odds of being a smoker are lower by a factor of 0.892 (ranges from 0.876 to 0.907); the likelihood of smoking decreases by a factor of 0.364 for those with a tertiary education as opposed to people without A-levels (ranges from 0.296 to 0.446); married people are 0.873 times less likely to smoke than single persons (ranges from 0.781 to 0.975); the odds of smoking are higher for females by 0.734 times (ranges from 0.676 to 0.796); and employees are 1.833 times more likely to smoke than non-employed persons (includes: retired persons, pupils, students, etc.: ranges from 1.673 to 2.007). Rows with no values are defined as reference categories.

Further, the t-test results verify the importance of the constraint “education” (i.e. the highest level of education) and “age” seen in the binary logistic regression. All other constraints are not applicable for the t-test because the dependent variable needs to be measured on a continuous scale (i.e. it is measured at the interval or ratio level). In this study, the level of education ranges from “university degree” to “no A-level degree”, whereas the independent variable “daily smokers”, consist of two groups: “daily smokers” and “never or never smoked daily”. Results from the t-test show that daily smokers are statistically significant for lower educated (2.80 ± 0.476) compared to “never or never smoked daily people” (2.71 ± 0.585). Further the study found that daily smokers are statistically significant for younger persons (5.62 ± 2.88) compared to people who “never” or “never smoked daily” (7.20 ± 3.36).

The census dataset from 2001 and the register-based census dataset from 2011 include demographic and socio-economic variables for people aged 15 and above at the municipality level, but no health related data. As mentioned above, these constraints must be available in both datasets at the municipality level, as well as comparable with the survey dataset so that the deterministic reweighting methodology can be applied and the output gained from the model will be as accurate as possible [9]. Table 3 shows the demographic change between the populations for both datasets for each sub-constraint. It can be seen that the constraint ‘age’ (with its sub-constraints 30–34 and 35–39) shows a strong decrease of individuals in that category, whereas other sub-constraints such as 45–49, or university graduates, show a strong increase of individuals over the 10 year period.

**Table 3:** Comparison of all constraints of all small areas between 2001 and 2011 (age 15+)

Constraint	Sub-constraint	2001	2011	Difference in %
Age	15–19	483,957	488,818	0.99
	20–24	472,777	527,675	10.40
	25–29	539,031	552,783	2.49
	30–34	668,281	538,307	−24.14
	35–39	704,872	564,817	−24.80
	40–44	625,783	675,242	7.32
	45–49	525,207	710,388	26.07
	50–54	514,535	626,162	17.83
	55–59	452,265	517,280	12.57
	60–64	451,057	480,665	6.16
	65 above	1,241,679	1,492,113	16.78
Education	University	497,754	831,629	40.15
	With A-level	763,430	976,652	21.83
	Without A-level	5,418,260	5,365,969	−0.97
Marital status	Single	2,060,472	2,400,266	14.16
	Married	3,527,786	3,562,949	0.99
	Widowed	573,318	573,070	−0.04
	Divorced	517,868	637,965	18.83
Sex	Male	3,195,725	3,465,023	7.77
	Female	3,483,719	3,709,227	6.08
Occupational status	Employees	3,541,877	3,801,016	6.82
	Employer	418,383	453,728	7.79
	Non-employed	2,719,184	2,919,506	6.86
Total population		6,679,444	7,174,250	6.90

For our final model smokeSALUD five constraint variables consisting of 23 sub-constraints (as seen in Table 3) are defined: education (3 sub-constraints), sex (2 sub-constraints), age (11 sub-constraints), marital status (4 sub-constraints), and occupational status (3 sub-constraints).

### Step 3: simSALUD model execution

**Fig. 2:**
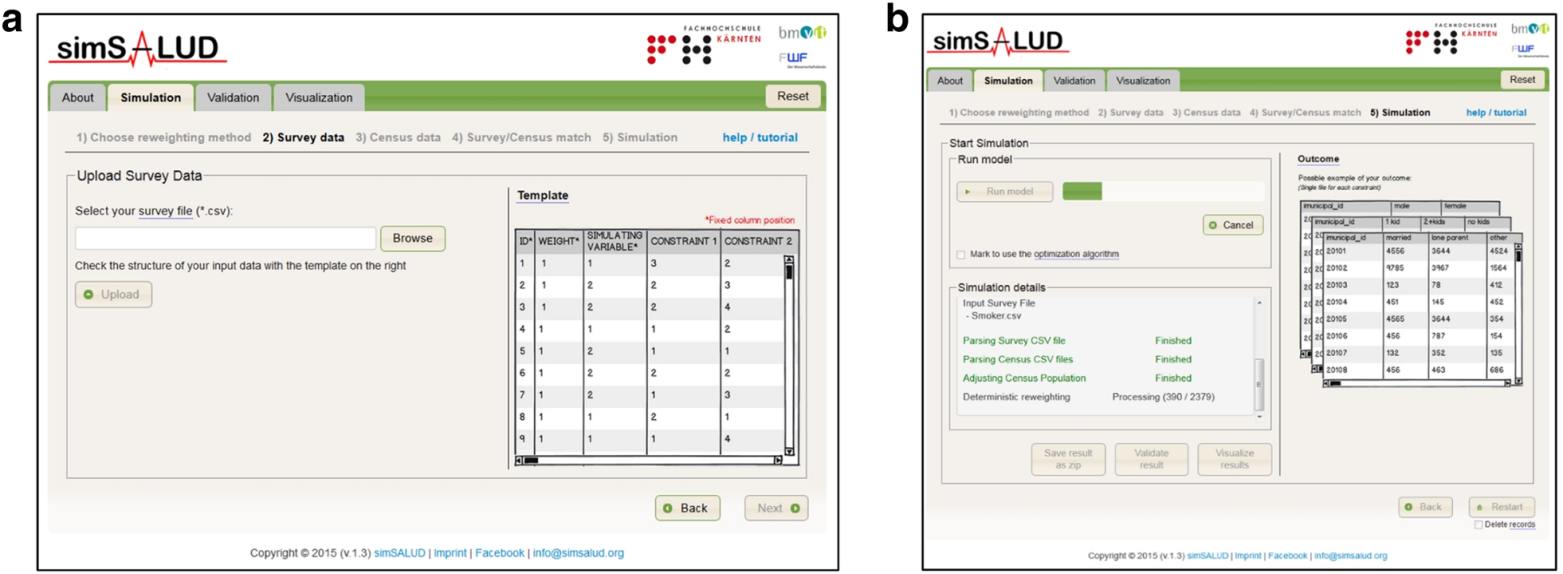
Screenshots of the simSALUD application. **a** The upload page for the survey file, **b** the page where the user starts the simulation

The first algorithm implemented within simSALUD is a deterministic combinatorial optimisation reweighting approach [2, 4, 14, 19, 20], combining (non-spatial) national survey data (AHIS 2006/07) with the (spatial) census data. Model 1 uses the census data from the year 2001 based on the constraint variables selected in step 2 (age, education, marital status, sex, occupational status). The simulation for model 2 uses the same constraint variables but the population data is taken from the registered-based census data for 2011. In contrast to a probabilistic approach, this reweighing technique is an iterative process with no random sampling where the ordering of the constraints is not an issue [21]. Within the simSALUD application, the prepared.csv files can be uploaded and the simulation steps are guided through a wizard for user-friendly software handling (see Fig. 2a, b). Additionally, the application has an integerisation method implemented after [19] to only allocate whole people to the simulation output. The results of the simulation can also be exported after the model run for further spatial analyses in common geographic information software (GIS) products.

### Step 4: Validation

After executing the model, the results are validated to ensure the model estimates are robust [22]. Distinguish between external and internal validation. External validation requires additional comparable external data sources which are usually not available, as the point of the simulation is to estimate ‘missing data’. This was unfortunately also the case for this study. There are however exceptions to the norm. For example, the New Zealand census includes data on the number of smokers for small geographical areas and this has proved to be a great source to validate simulated data on smoking patterns and rates see [3]. This type of study has helped to show the validity and robustness of small-area estimation techniques.

**Fig. 3:**
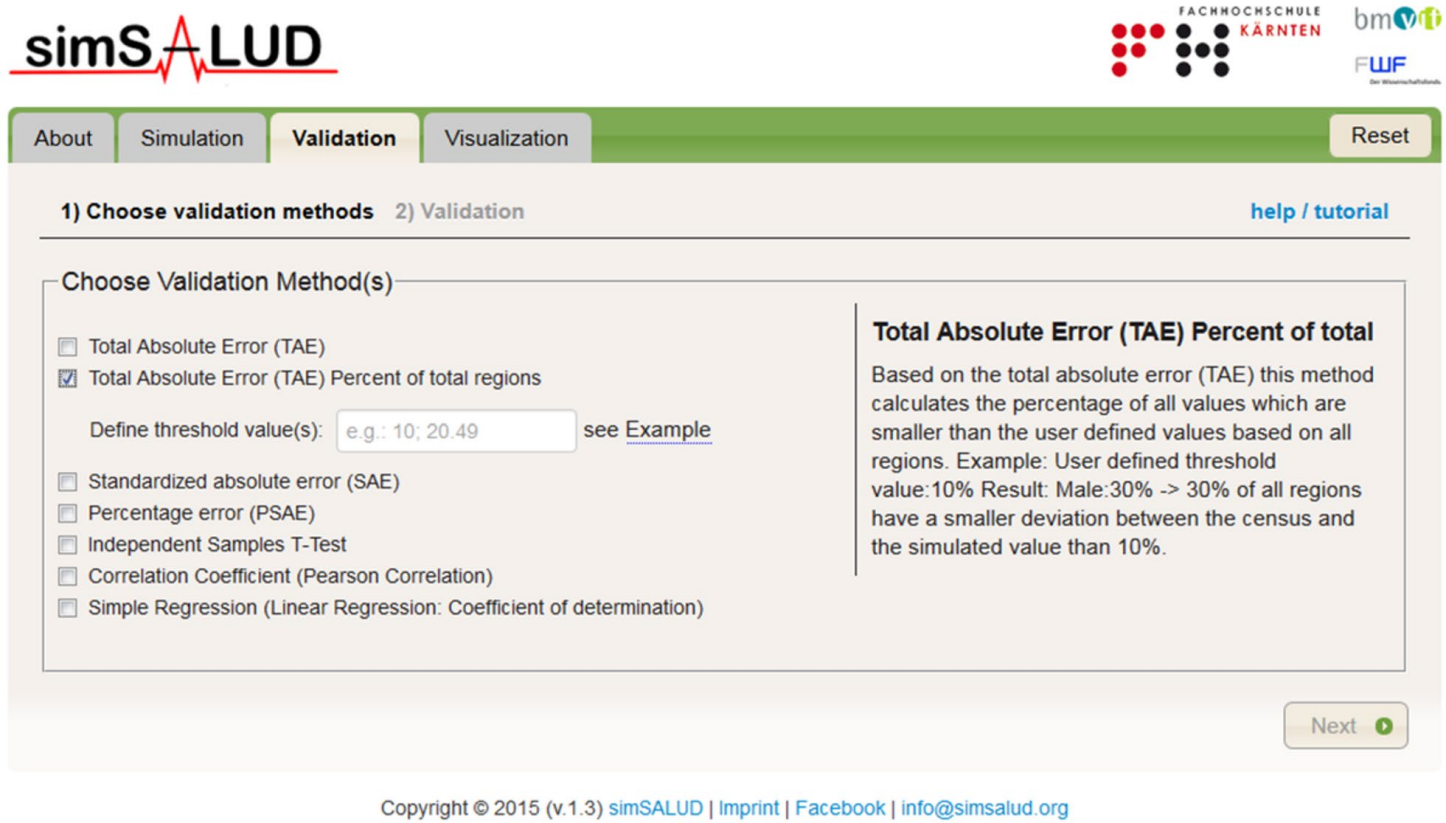
Screenshots of the simSALUD application which shows the menu to select the desired validation method

Internal validation refers to the ability of the model to replicate census data estimated at the individual level to spatial scales where the data is available. For internal validation, different statistical methods are offered within this Web application to achieve this. Currently, seven tests (see Fig. 3) are implemented from which the user can choose from, i.e. total absolute error (TAE), total absolute error with percentage of total regions, standardized absolute error (SAE), percentage standardized absolute error (PSAE), independent samples t-test, correlation coefficient (Pearson correlation) and simple regression.

This is a major advantage of simSALUD as the modeller does not have to use an additional statistics package and, further, he/she can map the validation results immediately (see Step 5: Visualization). After finishing the validation run the user can also either save the results as a zip file for further analyses or visualize the results immediately in the application (or transfer to a common GIS package). Additionally the user can start a new simulation and validation by changing the number of constraints to see the influence that each constraint can have on the simulated results.

### Step 5: Visualization

**Fig. 4:**
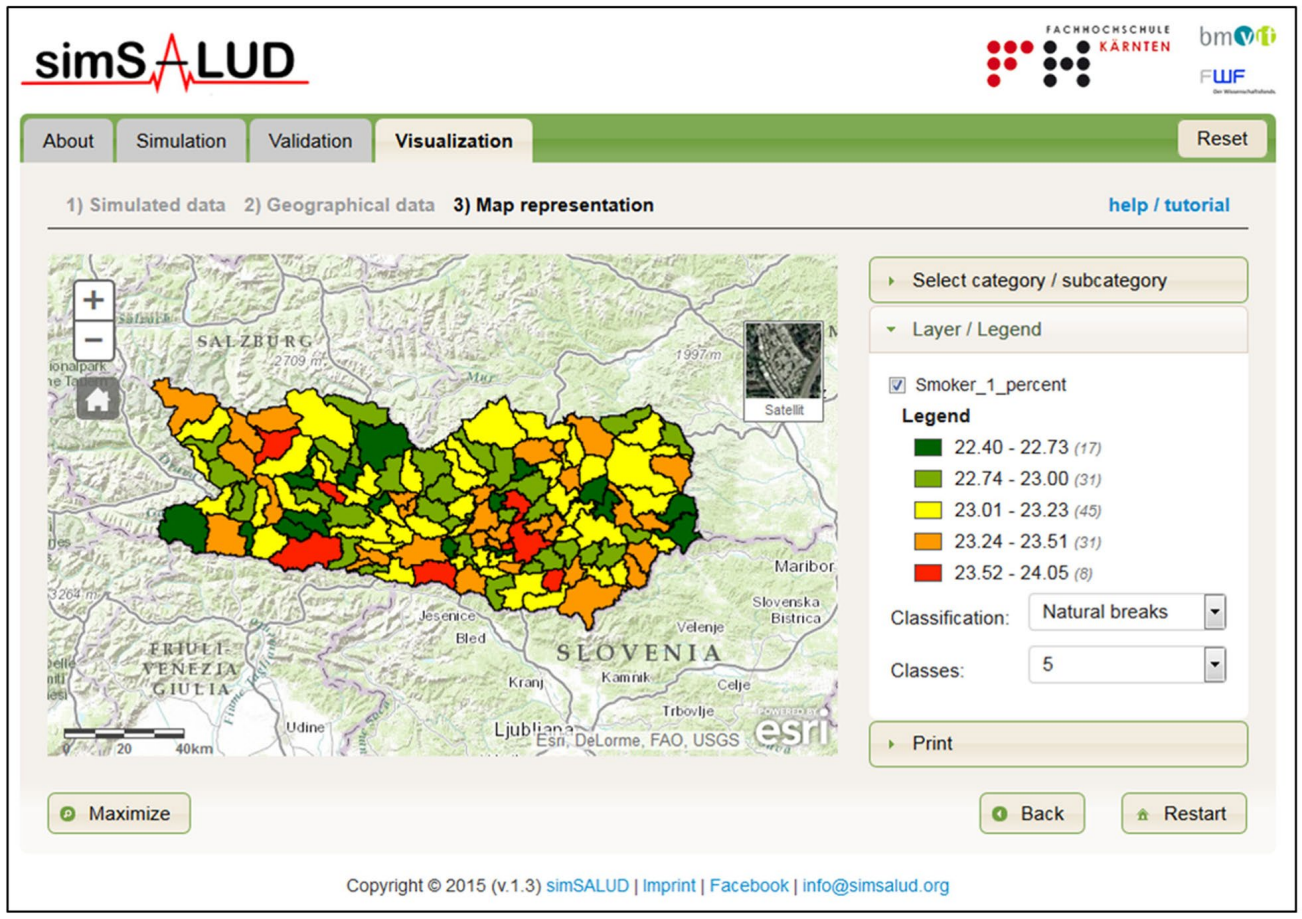
Visualization page of the application simSALUD

The simulation and validation outputs can be visualized in the form of a map (Fig. 4) and/or be linked with other data and external software for additional spatial analysis. The simSALUD Web-based application provides an interactive map with standard interaction controls that allows direct mapping without additional Geoinformation software or printouts. The map representation was implemented with Environmental Systems Research Institute (ESRI)’s JavaScript API (Application Programming Interface) with ArcGIS for Server. All spatial and non-spatial data (input and simulation results) are stored in a post-GIS database. The map representation allows the simulation results to be visualised according to any selected constraint. Additionally, the application provides different classification methods, i.e. equal interval, quantile and natural breaks, for mapping the results. Additional features are the resizing (maximize and minimize) of the map and the print feature.

## Results

The spatial–temporal analysis estimates the change of smokers in Austria from 2001 and 2011. The following two subsections describe the model validation results, as well as the estimated smoking prevalence illustrated as a map.

### Model validation

Model 1 (matching the 2001 census data with the AHIS 2006/07) and model 2 (matching the 2011 registered-based census data with the AHIS 2006/07) are validated using internal statistical tests. Table 4 shows the results of the PSAE and the linear regression for all sub-constraints. The PSAE shows the total under- or over-estimated values in comparison to the actual values for all sub-constraints. For 2001, the difference range from 0.4 % (sub-constraint: employer) to 6.9 % (sub-constraint: employees and non-employed) of the total population of each area. In 2011, the lowest value is 0.4 % (sub-constraint: employer) and the highest value is 8.0 % (sub-constraint: 65 above). A higher value shows a greater difference between the simulated and actual data for a particular sub-constraint.

**Table 4:** Summary of the validated model outputs for all constraints between 2001 and 2011

Constraint	Sub-constraint	PSAE		R^2^	
		2001	2011	2001	2011
Age	15–19	0.87	0.77	0.988	0.992
	20–24	0.85	1.48	0.993	0.993
	25–29	2.57	3.76	0.991	0.988
	30–34	2.38	2.21	0.999	0.999
	35–39	1.95	1.87	0.999	0.998
	40–44	0.86	1.07	0.998	0.997
	45–49	0.63	1.01	0.998	0.996
	50–54	0.52	0.66	0.996	0.992
	55–59	0.95	1.15	0.996	0.990
	60–64	1.93	2.12	0.993	0.988
	65 above	6.17	8.01	0.984	0.977
Education	University	3.38	4.36	0.997	0.997
	With A-level	3.18	3.39	0.998	0.997
	Without A-level	6.56	7.75	0.998	0.996
Marital status	Single	5.11	4.55	0.998	0.997
	Married	2.25	2.26	0.999	0.997
	Widowed	3.49	3.11	0.973	0.962
	Divorced	0.59	0.84	0.999	0.995
Sex	Male	4.91	4.81	0.999	0.999
	Female	4.91	4.81	0.999	0.999
Occupational status	Employees	6.90	6.68	0.999	0.9989
	Employer	0.39	0.42	0.997	0.9958
	Non-employed	6.85	6.93	0.996	0.9956

**Fig. 5:**
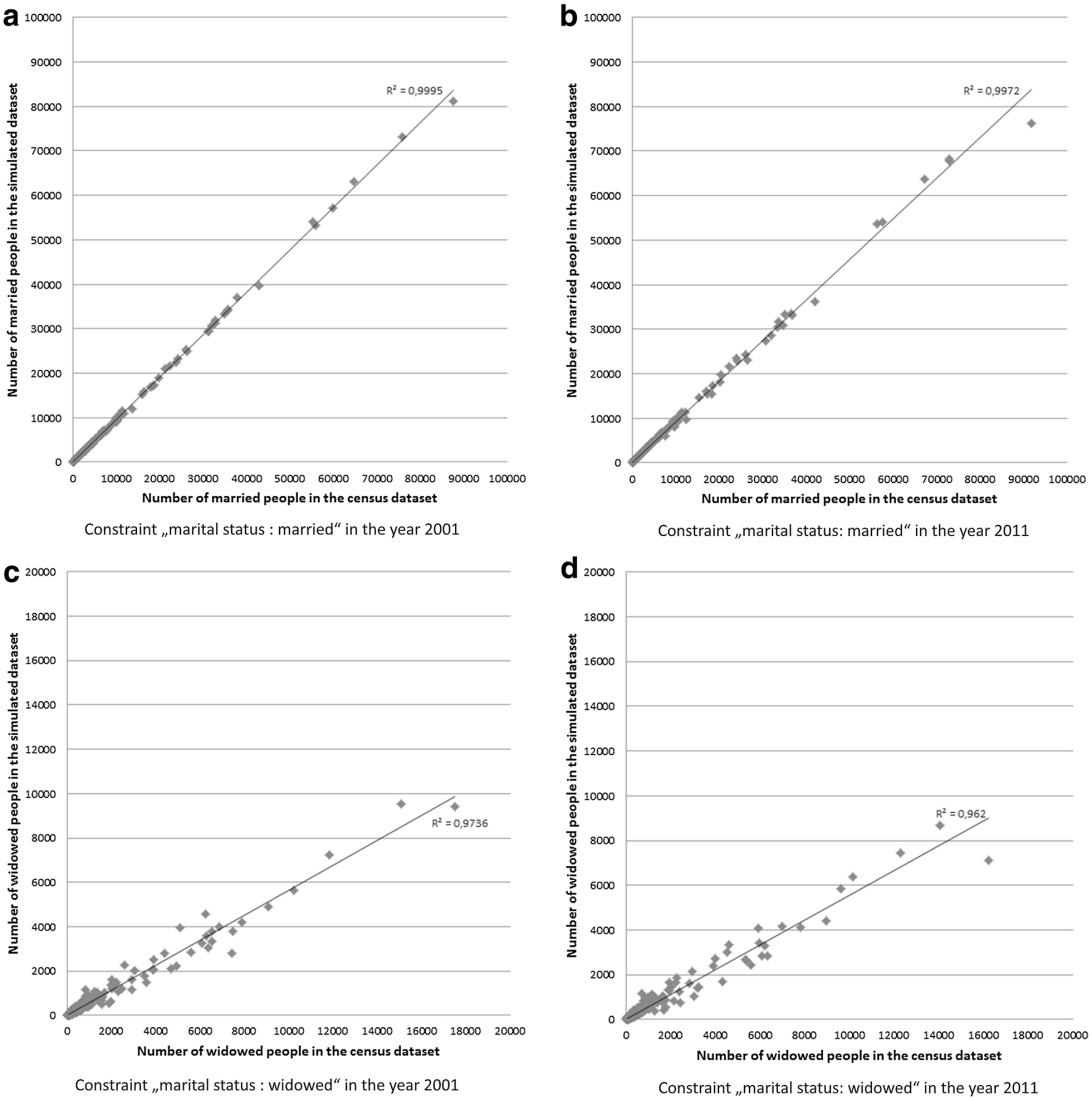
Validation results as linear regression models (comparing census and simulated data) for two selected constraints in 2001 and 2011, **a** married 2001, **b** married 2011, **c **widowed 2001, **d** widowed 2011

 The linear regression analyses for all constraint variables show a good fit, as an ideal fit between the simulated and census data would show a coefficient of determination very close to 1. This analysis indicates a very high coefficient of determination for all variables (0.96–0.99) for 2001 and 2011. The lowest value (with 0.96) is seen for the sub-constraints “widowed” and “age 65 above”. The variable “married” has a high coefficient for 2001 (Fig. 5a) and 2011 (Fig. 5b), where the simulated values are very close to the actual census dataset in comparison to the variable “widowed” where there is a higher variance between the simulated and the actual census data for both years 2001 (Fig. 5c) and 2011 (Fig. 5d).

Other possible constraints to predict smokers, for example “nationality”, are tested but did not show a significant improvement in the fit of the model. If the constraints fit well after the pre-analyses and validation procedure, then we can be confident that the simulated health variable (in this case “daily smokers”) also fits well and the results are robust.

### Estimated smoking prevalence

**Fig. 6:**
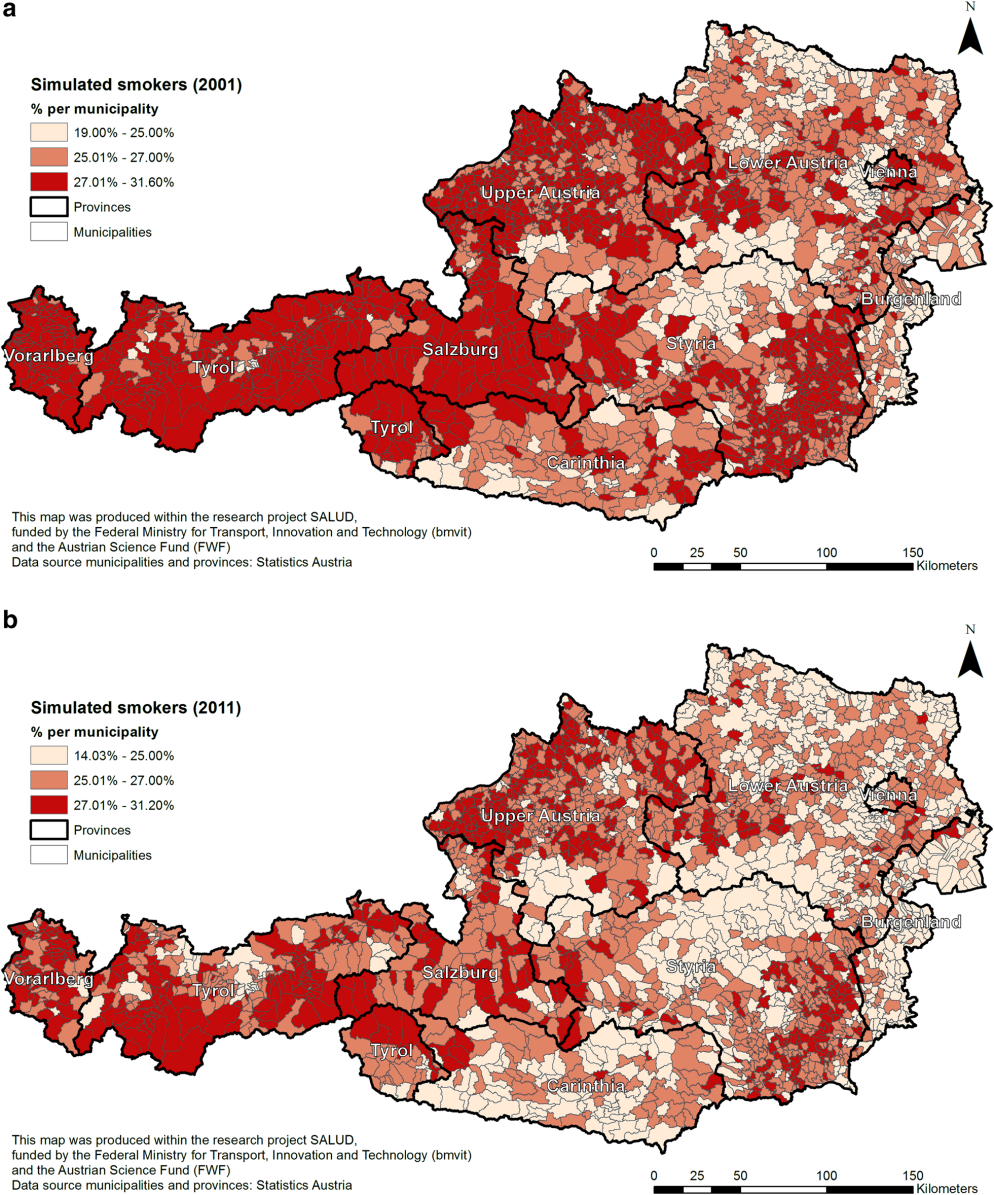
Simulated smoking population in Austria, **a** in 2001 and **b** in 2011

After running the simulation model for 2001, smoking rates between 19.0 and 31.6 % were estimated and for 2011, between 14.0 and 31.2 % (at the municipality level) (Fig. 6a, b). It was found that the average number of people who smoke slightly decreased over time (25.4 % in 2001 and 24.7 % in 2011) based on demographic change. The province Burgenland in the east of Austria shows lower smoking rates as expected, therefore the model underestimates for this province, and after discussions with governmental tobacco control groups, reasons are not fully explained yet as to while Burgenland had such a high smoking rate in the national survey 2006/07. The geography of the results show lower smoking rates in provinces with a higher proportion of rural areas. A decrease of smoking rates in the more rural municipalities can be seen particularly in Carinthia, Styria and Lower Austria. This is likely to an increase of elderly, married or widowed, people, as it is known that birth rates reduce, young people are moving into cities and past tobacco control was not strongly availably and effective, respectively.

**Fig. 7:**
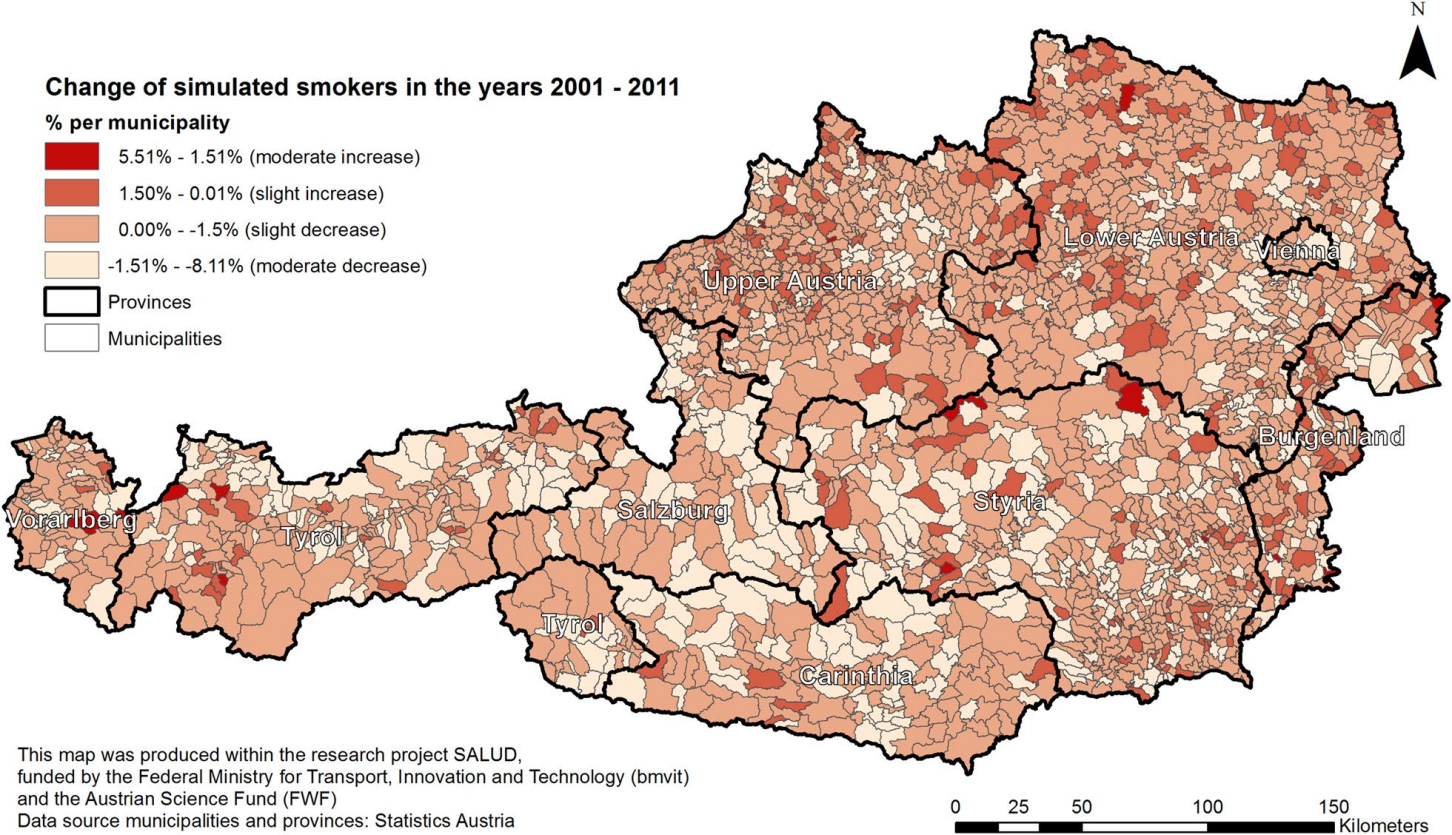
Spatial temporal change of smokers between the years 2001 and 2011

Figure 7 shows the spatial variation over time (between the years 2001 and 2011) for the number of estimated smokers. The map identifies a slight increase in the number of people smoking in some municipalities (with the highest values in northern parts of Styria and West Tyrol when aggregating from municipality level), but the vast majority of areas (especially in the northern parts of Carinthia and the south of Salzburg) show a decrease in the number of smokers between 2001 and 2011 (see brighter coloured municipalities). An interesting fact is that the municipalities with the highest increase (Namlos: 5.51 %) and highest decrease (Unterperfuss: 8.11 %) are both located in the province of Tyrol. Overall, the slight decrease in daily smokers based on demographic change could be due to the increase in the number of well-educated people, as education is a significant (negative) predictor of being a smoker. Further, population aging is likely to be another main reason, as there is an increase of elderly persons over time and the elderly are less likely to smoke. Both models show a west-east divide in smoking behaviour. To note is the decrease for all municipalities in Salzburg, when only with a very minor percentage. Further investigations in collaboration with governmental health departments could help to explore other possible reasons for higher and lower smoking rates in certain areas and what regional interventions these regions might benefit most.

## Discussion

A limitation of the *smokeSALUD* model itself is the availability of the same variables between the census data for both 2001 and 2011. Not all variables are the same between the two data collection periods (e.g. different subclasses) and therefore restrictions have to be accepted. The reasons for the mismatch might include the change in the survey method (from paper survey to electronic registered-based survey). However, Statistics Austria plans that in future the registered-based census for certain variables will be published annually, which will be an advantage for future models. Also the forthcoming AHIS will provide further interesting results in terms of changes in the size of the smoking population.

This study also found that the choice and number of constraint variables strongly impact the simulation results for the currently implemented static deterministic reweighting approaches. The advantage of such approach is that the impact of changes relating to input variables can be immediately measured, as it is a deterministic approach. For example, does the constraint variable “nationality” have a positive or negative influence on the model output? Therefore, several tests to find the optimal model of *smokeSALUD* were conducted. Also experiments with the sub-constraints were undertaken and it was found that different age groups did not have a great impact and therefore all eleven age groups were modelled. It is important to use variables that are very strong predictors and significantly correlated with the variable being estimated. Unfortunately the number of census constraints is limited in Austria: for instance, income could be an important predictor variable, but this data is available in the health survey data only and not in the census data, and is therefore not suitable to include in the model.

The model results could also benefit from additional external model validation, in addition to internal validation. Nationwide, there is no data on smoking prevalence for municipalities available, but alternatives could include carrying out a survey for some municipalities and potentially adjusting the model based on the results. Also, there might exist such data within an independent governmental survey for a particular region in Austria. However, the availability of such surveys is not known to the authors.

In spatial modelling, the modified area unit problem (MAUP) is a commonly discussed issue, as it can affect the outcome depending on the spatial unit chosen. The advantage of spatial microsimulation modelling is to work with individual data that will be matched to the chosen small area level unit based on common constraints. This means that individuals that have the same constraints are allocated to the small areas. The MAUP therefore have very little effect in this case (see further discussion in [23, 24]).

In an international context, the application simSALUD is freely available to anyone and this means people from all over the world can access or install it on their machines. Further, simSALUD is not limited to health studies only, as the underlying algorithms can be applied in other disciplines, such as economics or environmental science. A main advantage of simSALUD for smoking is that it offers a framework for testing the impact of smoking policies in different parts of the world. Using simSALUD policy makers could estimate smoking rates prior to the implementation of any policy and then again some years after its introduction. Although policy makers will know national responses to the policy (in terms of the overall numbers of persons quitting smoking) they can use simSALUD to estimate small-area changes and hence allow them to rethink strategies in areas which seemed to have resisted change the most. For these regions other policies may be more appropriate, such as increasing the price of tobacco or adding more stop-smoking clinics. Thus, it is hoped that simSALUD can be implemented worldwide in the future and not just used for Austrian data. For example, simSALUD is currently being tested in New Zealand using New Zealand census and health survey data.

Regarding policy, spatial microsimulation is very powerful as it allows to spatially model ‘what-if’ scenarios to identify the impact that certain interventions could have on certain population groups. Also important is the identification of areas most likely to expect highest and lowest impact of a specific intervention. An interesting future research project would be to take successfully proven interventions in other countries and apply these changes in a host country using simSALUD. For example what is the impact on the number of smokers when raising the legal age of purchasing cigarettes from 16 to 18 years and which areas would benefit most and least from this intervention (based on a region’s age profile)? Further steps could then be to link this analysis to economic factors to identify the impact of the costs that this intervention involves in relation to the number of smokers quitting (a type of cost-benefit analysis). The latter is especially interesting in identifying most beneficial interventions given available public health budgets. simSALUD not only allows us to specify particular population groups that can be modelled, for example less educated women in the age of 16–24 who are regular smokers, but also to target specific regions that are of high concern of the government, especially in terms of reducing health inequalities.

## Conclusions and outlook

This case study shows the application of *smokeSALUD* for modelling the spatial–temporal changes in the smoking population in Austria between 2001 and 2011. This is important as Austria has one of the highest smoking rates in Europe and no data on smoking exists at the municipality level. Policy makers can benefit from such data and spatial analysis in the future to help target specific areas of high need and hence try and reduce smoking prevalence. To build the model a deterministic reweighting algorithm was used that was implemented within the application simSALUD. This spatial simulation model matched data from the AHIS 2006/07 and the census and registered-based census data for the years 2001 and 2011 respectively. Results showed that there was a slight decrease in smoking rates of 0.6 % between the decades. This decline is thought to have been driven by the high increase (40 %) of those in tertiary education who are less likely to smoke and by the aging population, as older people are less likely to smoke. In particular, the province of Salzburg shows the highest decrease (1.6 %) of the smoking population whereas some municipalities in Tyrol show the highest increase. Not much change was seen in the existing west-east divide of the smoking prevalence. Regarding absolute numbers of smokers, all major cities have highest numbers in comparison to rural areas. This can be explained because of the high population size and contributes less to other factors. The findings of the study identified areas with slight and moderate increase of smoking prevalence and more research is needed to explore possible reasons for the increase, other than an increase in higher educated and elderly people. The model was validated using the statistical tests, the PSAE, logistic regression and the t-test, where the input constraints (census data) were compared with the simulated constraints. All three tests showed (for each model) acceptable and very good values (e.g.: high coefficients of determination) so that we can be confident that our model is robust.

The results presented in this paper are also being presented and discussed with various governmental health departments in Austria to highlight the importance of health data at the municipality level and the advantage of integrating this modelling algorithm into simSALUD. This package can help to support health care planners to identify variations in smoking rates within small areas, as such data is currently not available in health surveys. Further collaborative investigations could explore possible additional reasons for higher and lower smoking rates in certain municipalities. In addition, current policy initiatives around smoking prevention could be analysed and what-if scenarios for future modelling could be defined so that specific population groups are targeted. Possible links to the location of stop smoking services are of high interest: for instance, ‘how many smokers reside within a certain distance of stop smoking services?’ Where would be the optimal set of locations for the provision of such services? Non-spatial initiatives, for example increasing cigarettes prices, could also be tested to see if these would be effective, especially in localities with lower income residents (using any data which might be a good proxy for income)? see also [25, 26]. Such simulations and analysis, therefore, could give excellent support to health policy makers who are tasked to reduce smoking.

## Abbreviations

AHIS: Austrian Health Interview Survey
API: Application Programming Interface
CO: Combinatorial Optimization
ESRI: Environmental Systems Research Institute
EU: European Union
GIS: Geographic Information Software
IPF: Iterative Proportional Fitting
MAUP: Modified Area Unit Problem
OECD: Organisation for Economic Co-operation and Development
PSAE: Percentage Standardized Absolute Error
SAE: Standardized Absolute Error
SALUD: SpAtiaL SimUlation for Decision support
TAE: Total Absolute Error

## Footnotes

1
www.simsalud.org/simsalud.
